# Socially Extended Scientific Knowledge

**DOI:** 10.3389/fpsyg.2022.894738

**Published:** 2022-06-21

**Authors:** Duncan Pritchard

**Affiliations:** Department of Philosophy, University of California, Irvine, Irvine, CA, United States

**Keywords:** epistemology, social cognition, distributed cognition, extended cognition, virtue epistemology, scientific knowledge

## Abstract

A three-tiered account of social cognition is set out—along with the corresponding variety of social knowledge that results from this social cognition—and applied to the special case of scientific collaboration. The first tier is socially-facilitated cognition, which results in socially-facilitated knowledge. This is a form of cognition which, while genuinely social (in that social factors play an important explanatory role in producing the target cognitive success), falls short of socially extended cognition. The second tier is socially extended cognition, which generates socially extended knowledge. This form of cognition is social in the specific sense of the information-processing of other agents forms part of the socially extended cognitive process at issue. It is argued, however, that the core notion of socially extended cognition is individual in nature, in that the target cognitive success is significantly creditable to the socially extended cognitive agency of the individual. Socially extended cognition, in its core sense, thus generates individual knowledge. Finally, there is distributed cognition, which generates distributed knowledge. This is where the cognitive successes produced by a research team are attributable to a group agent rather than to individuals within the team. Accordingly, where this form of social cognition generates knowledge (distributed knowledge), the knowledge is irreducibly group knowledge. It is argued that by making clear this three-tiered structure of social scientific knowledge a *prima facie* challenge is posed for defenders of distributed scientific cognition and knowledge to explain why this form of social knowledge is being exhibited and not one of the two weaker (and metaphysically less demanding) forms of social knowledge.

## Introduction

Scientific inquiry is typically a collaborative endeavor in that it is most often conducted by close-knit groups of inquirers rather than a single individual. There is now a wealth of literature on scientific collaboration, and in particular on how such collective epistemic undertakings qualify as group activities that can produce group knowledge.^1^ I want to approach this issue via the specific lens of extended cognition, and therefore consider the extent to which the social activity of a highly collaborative scientific team constitutes a form of socially extended cognition, one that can produce positive epistemic outcomes (like knowledge). With this in mind, I will be arguing for a three-tiered conception of collaborative scientific knowledge. This approach thus leads to three ways in which we might understand “group knowledge” in this context.

On the weakest conception, which falls short of actual socially extended cognition, we have *socially-facilitated cognition* that leads to socially-facilitated scientific knowledge. Next, we have cases of *socially extended cognition* where nonetheless the collaborative scientific knowledge that results is individual knowledge on account of how the socially extended cognitive processes are individual rather than group cognitive processes. As I will explain, socially extended cognition is usually individual cognition of this kind. This type of collaborative scientific knowledge is not committed to there being irreducible group knowledge (i.e., group knowledge that is not reducible to the individual knowledge of the group members). Finally, there is a strong version of socially extended cognition---*distributed cognition*---whereby the socially extended cognitive processes belong to the group and not to individuals within the group.^2^ In this case the scientific knowledge that is generated by these processes is irreducible group knowledge, or *distributed knowledge*.

The point of this taxonomy is to show that one can capture at least two robust forms of social knowledge (and thus “group knowledge”) that apply to the scientific case that don’t bring with them the burden of being committed to irreducible group knowledge. In particular, conceiving of collaborative scientific knowledge as socially extended cognition that falls short of distributed cognition doesn’t entail irreducible group knowledge. Naturally, that doesn’t settle the question of whether there is irreducible group scientific knowledge (and this manuscript doesn’t attempt to settle that question), but it does put the onus on those who wish to argue for such a thesis to show that what is on display is not one of the two weaker forms of social knowing, particularly socially extended individual knowledge.

## Extended Epistemology

Let’s start with extended cognition. As is now familiar, this proposal holds that elements of an agent’s environment, such as instruments, can become, in the right conditions, proper parts of that agent’s cognitive processes. When that occurs, the agent is exhibiting an *extended cognitive process*, the supervenience base for which extends beyond the brain and central nervous system of the agent (indeed, typically beyond the skill and skull of the agent) to take in factors that are external to the agent.^3^ Moreover, when an extended cognitive process yields true beliefs, then we can ask what the epistemic standing of those beliefs are, and in particular whether they amount to knowledge. Call the knowledge that results from an extended cognitive process *extended knowledge*.^4^

Three qualifications are in order. First, I will not be defending extended cognition here, but rather taking this proposal as a given.^5^ Second, I will be restricting my attention to cognitive processes that generate beliefs as outputs. Clearly not all cognitive processes are of that kind. Some of them, for example, are entirely sub-personal, including in terms of the cognitive states that they produce.^6^ But these other cognitive processes, extended or otherwise, will not be our concern here. Finally, third, we will be specifically interested in extended cognitive processes that involve information-processing that is occurring in the agent’s environment. This qualification is important in that there are numerous ways in which features of the environment can arguably play the required role in extended cognitive processes that don’t involve information-processing, such as when, to take a familiar example, one uses one’s hands to aid one’s cognition.^7^ Such cases are interesting in their own right, but they will not be our concern here.

With extended cognition so construed, our question becomes whether there can be socially extended cognitive processes that lead to positive epistemic outputs like knowledge (i.e., *socially extended knowledge*). Insofar as this is the case, we will be considering what the criteria are for such processes and whether scientific collaborations satisfy them. Moreover, we will be interested in what kind of group scientific knowledge is entailed by thinking of collaborative scientific knowledge as socially extended cognition. Before we get to these issues, however, we should first consider the more familiar non-social case of technologically extended cognition, and thus the kind of extended knowledge that goes with this form of extended cognition.

Extended cognition is usually motivated by appeal to an individual’s use of technology, such as the famous case of “Otto” and his notebook offered by Clark and Chalmers (1998).^8^ Very roughly, Otto is a dementia sufferer who systematically employs his notebook in order to aid his memorial cognitive processes. Of course, not every instance in which a subject employs technology amounts to extended cognition, but the point about the Otto case, as Clark and Chalmers emphasize, is that Otto’s systematic employment of the notebook amounts to a kind of extended memorial cognitive process on account of how it is sufficiently on a par with his use of biological memory. This claim is encapsulated in their “parity principle”:

“If, as we confront some task, a part of the world functions as a process which, were it done in the head, we would have no hesitation in recognizing as part of the cognitive process, then that part of the world is […] part of the cognitive process.” (Clark and Chalmers, 1998, 29).

Accordingly, since Otto’s use of the notebook is (arguably, anyway) functionally on a par with his use of biological memory, so it ought to count as an extended memorial process whereby the notebook forms part of the extended cognitive process, as opposed to simply a case where Otto is employing the notebook merely as an instrument.

Of course, claims of parity of this kind are hard to assess. Given the number of factors in play, there are bound to be differences as well as similarities between the two cognitive processes (in this case between the use of the notebook and corresponding uses of biological memory). Which sub-set of these factors should we focus our attention on? In support of this parity principle, Clark (2010) helpfully offers the following set of criteria [which in turn make explicit criteria that are less explicitly invoked in Clark and Chalmers (1998)]:

(1) “That the resource be reliably available and typically invoked.”
(2) “That any information thus retrieved be more-or-less automatically endorsed. It should not usually be subject to critical scrutiny. […] It should be deemed about as trustworthy as something retrieved clearly from biological memory.”
(3) “That information contained in the resource should be easily accessible as and when required.” (Clark, 2010, 46)

Clark’s focus in offering these “glue and trust conditions”, as they are widely known, is explicitly the memorial case (in line with the original Otto example), but the idea is that such conditions are meant to be generally applicable, *mutatis mutandis*, to cases of extended cognition. The general idea is that the relevant standard to be applied in each case, in keeping with the parity principle, is that of the counterpart biological cognitive process. In line with the first condition, Otto’s notebook needs to be as reliably available and typically invoked as Otto’s biological memory (i.e., he carries around his notebook, and has it to hand when needed). In line with the second condition, the information in Otto’s notebook should be as automatically endorsed as his biological memories typically are, as presumably they will be given that they are his entries in the notebook (Note that this condition allows for some critical scrutiny of the entries in the notebook, just as we sometimes subject our biological memories to critical scrutiny). And in line with the third condition, the information in Otto’s notebook should be as readily accessible as his biological memories are, as presumably it will be, given that his notebook is to hand and the entries are easy accessible therein (Note that this condition is compatible with these entries not always being immediately accessible, given that one’s biological memories can sometimes be hard to recover).

Even if we grant that Otto’s use of the notebook satisfies the glue and trust conditions, it is still debatable that there is a genuine functional equivalence in play here regarding his use of the notebook and his comparative use of his biological memory. In particular, there doesn’t seem to be the kind of fluency involved in using a notebook, no matter how integrated it is with one’s cognitive processes, that compares with the fluidity of one’s use of biological memory. Of course, one could resist appeals to fluency of this kind and insist that there is sufficient functional equivalence to warrant treating this case as a genuine instance of extended cognition. But the worry is that lowering the bar for extended cognition in this way would be too permissive, in that it would lead to far too many uses of technology as counting as extended cognition.^9^ Nonetheless, even if Otto’s use of the notebook doesn’t quite make the grade as being a genuine case of extended cognition, once we understand what the criteria for extended cognition are we can imagine cases that fit the bill.^10^

In any case, wherever we eventually set the bar for genuine cases of extended cognition, insofar as such extended cognitive processes are successful in producing positive epistemic outcomes like true belief, then that true belief will be in the market for extended knowledge. Of course, it doesn’t follow from the fact that an extended cognitive process has generated a true belief that this true belief amounts to knowledge, any more than a non-extended cognitive process that generates a true belief has thereby generated knowledge. One can form true beliefs in all manner of ways such that they lack the epistemic credentials to make them knowledge. Still, there doesn’t seem any principled reason why the epistemic hurdle for extended knowledge should be any different from corresponding cases of non-extended knowledge.

Consider a prominent virtue-theoretic way of thinking about knowledge, such that knowledge is the result of the stable and reliable cognitive skills (epistemic virtues, broadly conceived) of the agent. In particular, on the virtue-theoretic model, one has knowledge when one’s cognitive success (=true belief) is at least significantly attributable to one’s manifestation of relevant cognitive agency (i.e., one’s display of relevant cognitive skill). Such a view can explain why, for example, mere reliable true belief won’t suffice for knowledge, as such reliability might not be significantly attributable to the subject’s cognitive agency. To take a famous case from the contemporary literature, if one’s reliable true belief is the result of a cognitive malfunction, such as a brain lesion, then that doesn’t amount to knowledge even if the true belief happens to be reliably formed.^11^

I’ve argued elsewhere that when this virtue-theoretic proposal is properly understood it only demands that the subject’s cognitive agency should be a *significant* part of the causal attribution of the subject’s cognitive success and not that it should be the *primary* or *overarching* element of such a causal attribution. This is important because it allows a virtue-theoretic treatment of knowledge to accommodate cases where the subject is epistemically piggy-backing off the epistemic contributions of others (or of information-processing technology). In testimonial cases, for example, one might gain knowledge by for the most part trusting the word of an expert. In such a case while one’s cognitive agency is playing a significant explanatory role in one’s cognitive success (one doesn’t gain testimonial knowledge by simply believing anyone, or whatever someone tells you; gullibility is not a route to knowledge), it is not playing the overarching explanatory role given the important contribution to the cognitive success made by the informant.^12^

This feature of the view can also allow us to accommodate technologically-facilitated knowledge. Subjects who appropriately employ technology when forming their true beliefs can still count as knowers, even despite the undoubted cognitive contribution of the information-processing technology that’s employed, so long as their own relevant cognitive agency is playing a relevant explanatory role in this cognitive success (Think, for example, of a skilled scientist using sophisticated information-processing equipment, such as an MRI brain scanner, in the laboratory).

What is interesting about this kind of virtue-theoretic way of thinking about knowledge, however, is that it is silent as to the nature of the cognitive skills of the subject, and in particular whether they are extended or non-extended. Accordingly, this way of thinking about knowledge is very amenable to understanding extended knowledge. All that matters is that the subject’s cognitive success is significantly attributable to her manifestation of relevant cognitive agency, where that might include both extended and non-extended cognitive processes. In the former case, we would thus have a virtue-theoretic account of extended knowledge.^13^

In particular, we now have a way of differentiating between knowledge that is merely technologically-facilitated and technologically extended knowledge. The issue ultimately comes down to what carries the explanatory load in terms of account for the cognitive success in question. In cases of technologically-facilitated knowledge, the subject’s onboard (unextended) cognitive processes are playing a significant explanatory role in that cognitive success, with the technology, construed as independent of this cognitive process, playing merely a supporting role. Normal use of instruments, including scientific instruments, will generate knowledge of this kind.

In contrast, in cases of technologically extended knowledge, the explanatory burden will be distributed differently. In particular, where a cognitive process is genuinely extended, the significant part of the explanatory burden will be attributable to that cognitive process. So, for example, where a scientist’s use of technology is sufficiently integrated into her cognitive practices such that it is as seamlessly employed as the corresponding on-board cognitive processes, then the cognitive success will become significantly attributable to that extended cognitive process. This is as opposed to that cognitive success being significantly attributable to the unextended cognitive process that is aided by technology that is independent of that cognitive process.

We can see this distinction in action by considering a concrete case. Consider the use of scientific instrumentation such as undertaking deep space mapping with the Hubble telescope. Skillfully using this technology, such that one is even in a position to gain knowledge through its employment, requires a high degree of expertise and background knowledge. For example, one needs to know how to use the instrument, to interpret the data one is receiving, to know how to adjust its direction, and so on. Assuming someone is skillfully employing this technology, we can then ask what kind of knowledge results. Clearly, initially at least, gaining true beliefs via the use of the Hubble telescope would simply be a form of technologically-facilitated cognition. One’s onboard cognitive processes would be playing a significant role in one’s cognitive success, with the technology itself merely playing an independent supporting role. The knowledge that results would thus be technologically-facilitated, but unextended, knowledge.

Suppose, however, that the transition to extended cognition is effected in this case. The subject’s use of the technology becomes highly integrated into her cognitive activities, with rich feedback loops forming between the use of the technology and her onboard cognitive processes, such that its employment becomes seamless and fluent (or, at least, as seamless and fluent as corresponding unextended cognitive processes).^14^ The subject’s relationship to the instrument would then plausibly no longer be one of subject and instrument but would be more akin to an extension of her onboard cognitive processes. We might find, for instance, that our scientist talks about “seeing” the cosmos through the use of Hubble telescope in just the same way that she talks about seeing her immediate environment, where this indicates how her use of the technology is now as fluent (or almost so) as using her eyesight to see things in the distance. In such a scenario, we would find that what bears the explanatory burden with regard to the subject’s cognitive success shifts accordingly. In particular, the significant explanatory burden will now be carried by the subject’s extended cognitive process that has integrated the use of the technology. The Hubble telescope is no longer viewed as an external instrument supporting the acquisition of unextended knowledge, but rather as a proper part of the subject’s extended cognitive processes. As a result, when the subject comes to know something via means of the instrument, this would no longer be non-extended knowledge that is facilitated by the use of an instrument, but technologically extended knowledge where the instrument forms a proper part of the extended cognitive process.

## Socially Extended Scientific Knowledge

With the foregoing in mind, let’s consider the prospects for a specifically socially extended cognition. The most straightforward way to understand this is as a sub-species of extended cognition, where the external factor that comprises the extended cognitive process specifically concerns the information-processing undertaken by other cognitive agents as opposed to technology.^15^ Socially extended knowledge would then be knowledge that arises from a socially extended cognitive process.^16^

As we’ve seen, when discussing technologically extended cognition, and thus technologically extended knowledge, we need to be alert to cases where the cognition is merely technologically-facilitated rather than being a genuine case of extended cognition (and thus where the knowledge that results is merely technologically-facilitated as opposed to being technologically extended knowledge). A parallel issue arises with regard to socially extended cognitive processes, in that we need to distinguish between merely socially-facilitated cognitive processes (which aren’t yet cases of socially extended cognition) and socially extended cognition proper. As with technological extended cognition we can express this point at the level of knowledge and thus distinguish between merely socially-facilitated knowledge and socially extended knowledge.

For example, there will be familiar cases of testimonial knowledge in the scientific case where one is appropriately forming one’s testimonial belief by trusting the word of an informant with relevant expertise. Consider a case where a subject is undertaking experiments in the laboratory and appropriately relying on a member of the scientific team to report results that are feeding into an experiment, such as important calibration readings of the equipment being used. Suppose our agent forms a true belief as a consequence concerning one of these calibration readings. As previously noted, that one is relying on the word of another doesn’t in itself prevent one from gaining knowledge. What matters is rather whether one is appropriately forming one’s belief in this case. With the scenario as it is described, one would expect that this belief is appropriately formed, in that the subject has an adequate rational basis for trusting this informant (she knows their credentials, she is aware of their track-record, etc.), and would also be suitably alert to obvious errors in the results that they report, given that she is familiar with the process of running such calibration readings. Accordingly, while the informant is undoubtedly carrying some of the explanatory load in terms of accounting for our subject’s cognitive success, this doesn’t prevent the subject’s own cognitive agency from playing a significant part in this regard too, and thus allowing her to gain knowledge.

The foregoing is clearly not a case of socially extended cognition, however, and hence is not a case of socially extended knowledge either. It is rather just testimonial knowledge (a general kind of socially-facilitated knowledge), albeit occurring in a scientific context. Just as one can gain knowledge in ways that are assisted by information-processing technology, so one can gain knowledge in ways that are assisted by the information-processing of others. But just as not every epistemic use of an instrument meets the criteria for technologically extended cognition, so not every epistemic use of an informant meets the criteria for socially extended cognition. Where those criteria are met, however, then the attribution relations concerning the target cognitive success alter accordingly. In particular, whereas previously the information-processing done by the informant plays an explanatory role that is independent of that played by the cognitive process, now it will be a proper part of a socially extended cognitive process, and hence that socially extended cognitive process will carry a larger explanatory burden with regard to the subject’s cognitive success. Where the true belief so formed amounts to knowledge, this would thus be socially extended knowledge.

Consider a variation on the previous case by way of illustration. This time, instead of the subject simply making use of her informant’s testimony as part of her cognitive projects, we build in a more systematic cognitive relationship between the two agents. Imagine, for example, that the scientific team at work in this laboratory have worked closely together, and in an organized fashion, over long periods. We can imagine rich feedback loops forming between the two collaborators and consequently their respective information-processing becoming highly integrated within their cognitive practices, with a deep trust of each other as sources of information arising as a result. Such a process would be akin to the kind of cognitive integration of an instrument that we saw at work in cases of technologically extended cognition, whereby instead of the use of the instrument becoming a seamless part of the subject’s cognitive processes, it is instead the collaboration with the colleague within this specific scientific context^17^. With the case so described it would become plausible to attribute the subject’s cognitive success to a socially extended cognitive process where the subject’s collaborator is now a proper part of this cognitive process. As a result, this socially extended cognitive process can generate socially extended knowledge.^18^

We thus have a distinction between two kinds of social scientific knowledge. In the first case, social scientific knowledge is simply individual knowledge that is socially facilitated in the sense that an important part of the explanatory burden of the target cognitive success is played by the social setting that the subject occupies. This is still a *bona fide* form of social, or group, knowledge, given that the social setting is playing this explanatory role in the target cognitive success, but it does not involve socially extended cognition and thus does not generate socially extended knowledge. In the second case, in contrast, we do have a genuine case of socially extended cognition, and thus socially extended knowledge is generated. This is thus a stronger form of social knowledge, in that the collaborative element of the social dynamic has become integrated within the subject’s cognitive processes to become a socially extended cognitive process, rather than a non-socially extended cognitive process that is merely aided by the collaboration in play.

Before moving on to consider a third level of social scientific knowledge, I want to consider an objection to socially extended cognition, and thus to socially extended knowledge, from Wray (2018). While Wray accepts that there can be technologically extended cognition, he believes that there is an inherent problem with socially extended cognition, at least to the extent that it is thought to generate socially extended knowledge. The issue relates to a disanalogy between the two forms of extended cognition, in that technologically extended cognition doesn’t involve a distinct cognitive agent in the way that socially extended cognition does. Wray thinks that this is problematic because of how having additional agents involved complicates the relations of causal attribution for cognitive success. In particular, where there are two agents involved, there will be a natural temptation, argues Wray, to apportion the credit for the cognitive success to both of the agents rather than to consider one of the agents as employing a socially extended cognitive process (with the result that the lion’s share for that agent’s cognitive success goes to that agent and her socially extended cognitive process).

Wray usefully illustrates this issue by considering cases of scientific collaboration where things go awry, such as where one of the agents involved did not fulfill her cognitive responsibilities appropriately (e.g., by falsifying evidence), albeit in ways that were not apparent to the other collaborators. The details of the cases that Wray offers are not important. What is important is that in such cases the agents involved naturally insist that the responsibility for the malpractice should be solely on the head of the individual researcher who committed the malpractice and not assigned to the scientific group itself. Wray believes that this shows that even in tightly integrated scientific teams, there are clear lines of cognitive responsibility that ultimately concern the individuals involved, even if those lines of cognitive responsibility are not always apparent.

Wray’s objection is very interesting, but I don’t think that it poses even a *prima facie* problem for the cases of socially extended cognition and knowledge that we have considered thus far (though as we will see in the next section it does pose a *prima facie* problem for distributed cognition and knowledge). The reason for this is that socially extended cognition, as we have so far described it anyway, is precisely concerned with an *individual* cognitive responsibility on the part of the cognitive subject. This is why individual knowledge is usually the result of the socially extended cognitive process. In maintaining that other agents are proper parts of the socially extended cognitive process in play, we are not thereby claiming that the agent isn’t individually cognitively responsible for her cognitive success (as it is her socially extended cognitive agency that is carrying the significant explanatory burden with regard to the cognitive success).

If that’s right, however, then one might be puzzled as to why there is any shift in the kind of responsibility taken for collaborative cognitive success in light of the scientific malpractice of a group member coming to light. I think that what this shows is that sometimes collaborators can be unaware of the true social nature of a cognitive process that they are engaging in. In particular, if one is participating in a tightly integrated scientific team where, unbeknownst to one (and in ways that are not easily detectable), one of the members of the team is (say) fabricating results, then what this entails is that this member of the team is not in fact playing a causal explanatory role in producing the relevant cognitive successes. That is, the team members might reasonably suppose that all members of the team are playing a part in producing this cognitive success, and thus that for some of the members of the team at least, their individual knowledge of team results involves a cognitive process that features other team members as proper parts. In fact, however, there is a team member whose contribution is playing no causal explanatory role with regard to the target cognitive success (Indeed, their causal explanatory role is likely to only be significant when it comes to explaining the cognitive *failures* of the group). With this in mind, what is occurring in the kinds of cases that Wray describes, where it comes to light that a team member is not playing the cognitive role that they were supposed to, is that there is a recalibration of where the cognitive responsibility lies. It doesn’t follow that that there was no socially extended cognition on display; only that there are fewer collaborators who are proper parts of the socially extended cognitive process than initially thought.

## Distributed Scientific Knowledge?

We have distinguished between two forms of social scientific knowledge, where only one of the two varieties involves genuine socially extended cognition. Notice, however, that the case of socially extended cognition that we have described, while a robust form of social scientific knowledge, nonetheless ordinarily results in individual knowledge. That is, while the scientific collaboration met the criteria for socially extended cognition, as the case was described this had the consequence of leading to the individual agent (or agents) concerned employing a socially extended cognitive process that included the collaboration with the other subject as a proper part. As a result, just as technologically extended cognition leads to the individual concerned gaining technologically extended knowledge, so a socially extended cognitive process leads to the individual concerned gaining socially extended knowledge.

This is significant, since we can at least conceive of a stronger form of socially extended process—*distributed cognition*—where the cognitive process is not attributable to an individual but to a group of agents. So understood, distributed cognition would generate a cognitive success that is attributable to the group as a whole *as opposed to* being attributable to individual members of the group (indeed, in the most interesting version of this case, *only* the group comes to have knowledge, with all the individual members of the group lacking this knowledge). Where this cognitive success amounts to knowledge, we would thus have a form of irreducible group knowledge: *distributed knowledge.* That a scientific collaboration involves socially extended cognition does not entail distributed cognition since, as we have seen, it is compatible with the individual agent possessing the socially extended cognitive process, and hence being individually credited with socially extended knowledge. In short, in granting that there is socially extended knowledge one is not obliged to also endorse distributed knowledge.

This distinction is especially important because of the metaphysical commitments involved in endorsing distributed knowledge, which go beyond the commitments required for socially extended knowledge. In particular, it seems that allowing for distributed knowledge entails a commitment to there being a group agent that is distinct from, in the sense of not being reducible to, the agents who are part of that grouping. For there to be distributed knowledge of this kind, after all, there would need to be a distributed knower, and also, given that knowledge entails belief, the group agent must be able to have beliefs too.^19^ In contrast, where we think of the knowledge possessed by the group as reducible to individual knowledge (as in the case of non-distributed socially extended cognition), then we wouldn’t incur any metaphysical commitment of this kind. For example, we might loosely talk of scientific collaborations of the two kinds noted above as ‘‘group’’ knowledge, but this wouldn’t carry any commitment to there being an actual group agent, since we could understand this locution as simply picking out a kind of social knowing that is ultimately attributable to individual agents of the group.^20^

With the foregoing in mind, are there reasons for insisting that collaborative scientific knowledge must be understood as distributed knowledge? I’ve argued elsewhere that one prominent (but usually non-scientific) example of group knowledge, involving transactive memory systems, is best understood as a kind of socially extended knowledge that falls short of distributed knowledge.^21^ But that obviously leaves it open that there might be scientific cases that nonetheless fit the bill. There are numerous studies of collaborative scientific knowledge in the literature, and many of them seem to at least suggest such a conclusion. For example, the sociologist Knorr-Cetina (1999) offers a celebrated discussion of the kind of scientific collaborations involved in High Energy Physics (HEP) experiments. This involves extremely large teams of researchers, often working in different locations, and a wide range of specialized expertise and technology. Knorr-Cetina claims that no individual has overall responsibility for these experiments, and that the experiments are instead managed via structures of communication within the group. This includes a complex grid of meetings between researchers, such as research and development meetings, steering group meetings, workshop meetings where results are disseminated, and even interactions of an informal nature, such as ‘‘meetings after the meeting’’ where colleagues discuss the project in a more casual manner. The knowledge produced by these activities thus looks very much like a kind of distributed group knowledge that is fundamentally distinct from the individual knowledge of the group members.^22^

The interesting question for our purposes, however, is whether such complex social structures require us to attribute distributed knowledge to a group agent—i.e., to treat the team of collaborators as a whole (or a subset thereof) as being an emergent cognitive, and thus knowing, agent, distinct from the individual cognitive agents that make up the research team. While I can see the temptation do so, I think that the framework we have offered for understanding social cognition and knowledge should give us cause for hesitation. For while, we can coherently talk of this group having knowledge, there are natural ways of understanding this claim in terms of the knowledge of the individuals within the group. Moreover, although it is undoubtedly true that much of this individual knowledge is highly dependent on the cognitive contributions of other members of the research team, that is quite compatible, as we have seen, with both socially-facilitated and non-distributed socially extended cognition (and thus with individual knowledge).

Indeed, I think that Wray’s objection to socially extended cognition and knowledge that we considered earlier is in fact better directed at the idea of distributed cognition and knowledge. As Wray notes, when there is scientific malpractice within a research team, it quickly becomes apparent where the individual lines of cognitive responsibility lie, which suggests that they were identifiable all along (even if not clearly demarcated or otherwise left implicit). As we noted above, this does not pose a problem for socially extended cognition and knowledge as this is ultimately attributable to an individual cognitive agent (And it obviously isn’t a problem for socially-facilitated cognition and knowledge). It does pose a challenge to distributed knowledge, however, since that is committed to there being distributed knowledge, and thus a group agent, that is distinct from the knowledge possessed by the individual members of the research team.

Of course, the foregoing doesn’t establish that all social scientific knowledge is to be understood as either socially-facilitated or non-distributed socially extended cognition. My goal is rather to show that once we are aware of these two weaker senses of social scientific knowledge, then the onus is squarely on defenders of distributed scientific cognition, and thus distributed scientific knowledge, to demonstrate that the cognitive processes on display have to be understood along these particularly robust lines and not in terms of the weaker kinds of social cognition that we have identified.

## Data Availability Statement

The original contributions presented in the study are included in the article/supplementary material, further inquiries can be directed to the corresponding author.

## Author Contributions

The author confirms being the sole contributor of this work and has approved it for publication.

## Conflict of Interest

The author declares that the research was conducted in the absence of any commercial or financial relationships that could be construed as a potential conflict of interest.

## Publisher’s Note

All claims expressed in this article are solely those of the authors and do not necessarily represent those of their affiliated organizations, or those of the publisher, the editors and the reviewers. Any product that may be evaluated in this article, or claim that may be made by its manufacturer, is not guaranteed or endorsed by the publisher.

## Footnotes

1 See, for example, Giere (1988, 2002), Thagard (1993, 1994, 1997, 1999), Knorr-Cetina (1999), Latour (1999), Giere and Moffat (2003), and Nersessian (2006).

2 It should be noted that distributed cognition is sometimes used in the literature to simply refer to socially extended or group cognition, rather than having the specific meaning just given to it. Nonetheless, as we will see, it will be useful to distinguish between socially extended cognition and distributed cognition in the way that we do here. Moreover, my use of this terminology, while not universal, is fairly common. See, for example, Giere (2007) and Palermos (2020).

3 The *locus classicus* in this regard is Clark and Chalmers (1998). For some other key works on extended cognition, see Wheeler (2005), Clark (2008), and Menary (2010).

4 For further discussion of the epistemological ramifications of extended cognition, and thus of the idea of extended knowledge, see Pritchard (2010, 2018a,2018b), Palermos (2011, 2014a), Carter (2013), Clark (2015), and Jarvis (2015). See also the manuscripts collected in Carter et al. (2018a).

5 For some of the key recent literature that critiques extended cognition, see Adams and Aizawa (2001, 2008), Rupert (2004, 2009), and Sprevak (2009).

6 For an important recent discussion of the relevance of sub-personal cognitive processes in cases of extended cognition, including with regard to the epistemological ramifications of extended cognition, see Clark (2015). For more on the epistemological relevance of subpersonal cognitive processes, see Carter and Rupert (2020).

7 One important variety of extended cognition where the extended cognitive processes do not employ information-processing features of the environment is *embodied cognition*—see Noë (2004), Gallagher (2005), Chemero (2009), Rowlands (2009), and Shapiro (2011). For discussion of the specific instance of embodied cognition involving the free use of one’s hands when performing certain cognitive tasks, see Clark (2013), Wheeler (2013), and Pouw et al. (2014).

8 Note that this case, along with the manuscript in which the case appears, was specifically meant to be motivating the extended mind thesis rather than the extended cognition thesis that we are considering here. This is the claim that a subject’s mental states can supervene on features of that’s subject’s environment, such that, for example, a subject’s beliefs can have an extended supervenience base. Whether the extended mind thesis can be disentangled from the extended cognition thesis is a complex issue that we can set aside here. For a very useful recent overview of the extended mind/cognition theses, and how best to understand them, see Sprevak (2019).

9 This is the so-called “cognitive bloat” objection to extended cognition. See, for example, Sprevak (2009).

10 Consider, for example, the kind of *neuromedia* described in Lynch (2014) and Pritchard (2018c), where the technology is so embedded in one’s on-board cognitive processes that one is often not even aware, at the time of use, that one is employing an extended cognitive process. Of course, it goes without saying that no existing technology might qualify as neuromedia, but the point is just that such a technology is possible. Interestingly, while neuromedia does involve extended cognition, the cognitive augmentation in question might well be under the skin and skull of the subject, and in this sense “internal” (indeed, there might be good design reasons for this, as it would likely make it easier to integrate it within one’s on-board cognitive processes). Nonetheless, it would still be “external” in the relevant sense (i.e., external to the subject’s brain and central nervous system). For further discussion of the epistemology of cognitive enhancement, see Carter and Pritchard (2019).

11 The example is from Plantinga (1993, 195–98 & 205–207).

12 I’ve argued for this “modest” version of virtue epistemology in a number of places, but see, especially, Pritchard et al. (2010, ch. 3) and Pritchard (2012). For some of the key defenses of a stronger “robust” version of virtue epistemology whereby knowledge involves cognitive success that is primarily attributable to one’s cognitive agency, see Sosa (1991, 2007, 2009, 2015), Zagzebski (1996), and Greco (2010).

13 For more details about such a proposal, see Pritchard (2018b). Of course, while virtue epistemology can accommodate extended cognitive processes, it is undoubtedly the case that many contemporary epistemologists implicitly suppose that all cognition (of a kind that’s relevant to knowledge at any rate) is unextended. Sometimes this commitment is made explicit—see, for example, Goldman (1986, 51).

14 For further discussion of the notion of cognitive integration in play here, see Palermos (2014a,b) and Alfano and Skorburg (2016).

15 For some of the key texts on socially extended cognition, see Hutchins (1995), Giere and Moffat (2003), Giere (2006, 2007), Theiner et al. (2010), and Gallagher (2013).

16 For recent discussion of socially extended knowledge, see Carter et al. (2014), Lackey (2014), Wikforss (2014), Palermos and Pritchard (2016), Palermos (2020), and the manuscripts collected in Carter et al. (2018b).

17 For further discussion of cognitive integration in the specific context of socially extended cognitive processes, see Theiner et al. (2010) and Palermos (2016).

18 In the scenario as it is described one would expect both agents to develop socially extended cognitive processes. Note, however, that we can also have “mixed” cases of socially-facilitated and socially extended scientific knowledge within a specific scientific collaboration. This might arise, for example, in a two-person collaboration where the one subject’s information-processes becomes a proper part of the other subject’s cognitive processes but not *vice versa*.

19 Group belief, and group knowledge involving such group belief as a constituent propositional attitude, is defended in Tuomela (2004) and Gilbert (1987, 2002, 2004, 2009). See Schweikard and Schmid (2013) for a helpful overview of the issues in this regard. See also Tollefsen and Dale (2011). Another option in this regard (though also revisionary, albeit in a different way), is to argue that group knowledge is a distinctive kind of knowledge that is not attributable to a subject. See, for example, Giere (2007).

20 Most ways of understanding “group” knowledge are of this kind, in that what is being attributed to the subject is essentially reducible to the knowledge of the agents in that grouping (Similarly, many accounts of group “belief” treat them as essentially reducible to the beliefs, or at least the commitments in a broad sense, of the agents in that grouping). Note that the reduction might be relatively complex. For example, it is usually thought implausible that “group” knowledge in this loose sense should be understood as entailing that every member of the grouping has the knowledge (or belief) in question. For helpful discussion of the issues in this regard, see Bird (2019) and Tollefsen (2019). For discussion that is specific to the scientific case, see Bird (2017).

21 See Pritchard (Forthcoming). For some important discussions of transactional memory systems in the context of group and extended cognition, see Wegner et al. (1985), Wegner (1986), Sutton (2008), Sutton et al. (2008), Huebner (2016), Michaelian and Arango-Muñoz (2018), Michaelian and Sutton (2018), and Palermos (2020).

22 Knorr-Cetina (1999) contrasts the “communitarian” nature of HEP experiments with the more “individualistic” kind of scientific inquiry that she finds in her other main case study, involving molecular biology.
